# Subsequent Injury Risk Following Concussion in Australian Football League (AFL) Male Athletes: Is It a Case of a Player Being ‘Luckless’?

**DOI:** 10.3390/jfmk11020195

**Published:** 2026-05-14

**Authors:** Alan J. Pearce, Samuel Hardy, Kane Middleton, Doug King

**Affiliations:** 1 School of Health Sciences, Swinburne University of Technology, Melbourne 3122, Australia; 2 Independent Researcher, Melbourne 3000, Australia; 3 School of Allied Health, Human Services and Sport, La Trobe University, Melbourne 3086, Australia; k.middleton@latrobe.edu.au; 4 Sports Performance Research Institute New Zealand, Auckland University of Technology, Auckland 1142, New Zealand

**Keywords:** sports-related concussion, musculoskeletal injury, Australian rules football, professional athletes

## Abstract

**Background:** Sports-related concussion (SRC) is associated with elevated subsequent musculoskeletal (MSK) injury risk, yet no study has examined whether a mandatory post-concussion rest period mitigates this risk in professional Australian rules football. The aim of this study was to compare subsequent injury risk following SRC in Australian Football League (AFL) male athletes across two periods: a discretionary return-to-play period (2016 to 2019) and a mandatory 12-day non-competition period (2021 to 2024). **Methods:** Injury data were extracted from publicly available AFL injury reports across eight seasons (*n* = 4351 players). Risk ratios (RRs) with 95% confidence intervals were calculated using log-binomial methods, with pooled estimates derived via the Mantel–Haenszel approach. **Results:** Across both periods, SRC significantly increased overall MSK injury risk compared to MSK-first injury risk (2016 to 2019: RR:1.59 [95%CI:1.31 to 1.92]; *p* < 0.001; 2021 to 2024: RR:1.56 [95%CI:1.28 to 1.91]; *p* < 0.001). Upper and lower limb injury risks were similarly elevated across both eras. Notably, the risk of subsequent concussion was significantly elevated during 2016 to 2019 (RR:3.14, *p* < 0.001), whereas no statistically significant elevation was demonstrated during 2021 to 2024 (RR:1.30, *p* = 0.324). **Conclusions:** During the 12-day rest period, subsequent concussion risk was no longer significantly elevated, while MSK injury risk remained increased, suggesting longer recovery periods may be warranted for full neuromuscular recovery following SRC.

## 1. Introduction

Sports-related concussion (SRC) remains a notable and continuing international concern across contact- and collision-based sports. The Concussion in Sport Group (CISG) publishes recommendations for SRC definition and management and rehabilitation for return to competition [1]. The focus of management of SRC has been on initial symptom recovery, then through an active but graduated submaximal exercise rehabilitation approach to reduce the risk of persistent symptoms. The criteria for return to play (RTP) clearance are met via a clinical decision pathway involving the resolution of concussion-related symptoms and neurocognitive deficits, followed by remaining asymptomatic throughout the graduated rehabilitation pathway, to full contact training [1].

While the CISG recommendations are not specifically designed to reduce further risk of injury, for over a decade, there have been questions regarding the risk of subsequent injury after RTP in athletes who sustained a concussion [2]. Systematic reviews exploring this risk at professional and collegiate levels of sport and the military, have reported an increased risk of subsequent injury following a concussion. For example, the risk of subsequent lower-limb injury increases by over two-fold in both males and females [3,4], 1.8-fold for both sexes [4], and 2.2-fold in the upper limbs in male athletes [5]. McPherson et al. [6] also reported that anterior cruciate ligament (ACL) injuries were increased by two-fold in males following a concussion, but were only increased by 1.2-fold in females. Conversely, recent research showed that ACL injuries did not increase the risk of concussions [7], suggesting a directional risk reflecting neuromuscular disruption following concussion [8,9].

Despite the popularity of Australian football as a participation and spectator sport, little research has been conducted on the risk of subsequent injury after concussion. At the highest level, the Australian Football League (AFL) administrates the league at the professional level. While recent research reported that sporting performance appeared to be unaffected following concussion in professional AFL players [10], questions remain regarding the risk in this particular cohort. To date, there have only been two studies examining injury post-concussion, one in the AFL [11] and the other in a junior Australian football league [12]. In a 2009 study by Makdissi et al. [11] covering 158 players across four professional seasons (2000–2003), it was reported that the incidence of subsequent injury was elevated (incidence ratio 2.23 [95% CI: 0.93 to 5.04]) when compared to non-injured control players injuries per 100 games, respectively). However, as the 95% confidence interval crossed 1.0, the possibility of no true difference in risk could not be ruled out; therefore, the authors reported that this difference was not statistically meaningful. As opined by Bennett et al. [12], there have been many rule changes since that study that have led to increases in match intensity [13], and a greater general awareness of concussion recognition and management protocols that has contributed to an increase in reported concussions, which may influence consequent injury risk as seen in other sports.

The risk of further injury after concussion has also been investigated in an elite state-based Australian football junior league [12]. This prospective study collecting injury data from 2015 to 2022 showed no subsequent risk of injury after a concussion. The authors suggest that their findings may reflect the type of sport studied and differences between adult and junior athletes.

In 2021, the AFL was the first professional sporting league to implement a mandatory non-competitive period after concussion of 12 days [14]. The objective was to ensure proper management of concussed players, as there were concerns that players were being returned to competition too quickly. This became apparent following the introduction of a 2020 AFL policy requiring that medical clearance by the club doctor be completed at least five days prior to the team’s next scheduled competition match [15]. Despite the good intention of this policy, group level analysis by Pearce et al. [16] highlighted that this policy reduced the mean time to return to competition to 11.2 days, compared to the 2017–2019 seasons that identified a mean time to return to competition of 16.2 days (range 12.9–18.7 days). Further, a study by McCrea et al. [17] in US collegiate athletes comparing concussed athlete management from 1999–2001 and 2014–2017 showed that a generally longer and more conservative management in the latter period led to the reduction in recurrent concussion incidence.

The implementation of this policy provides us with a unique opportunity to analyse and compare the association between mandatory rest periods and further risk of injury. Therefore, the objective of this study was to compare subsequent risk of injury following concussion in AFL male players from 2016 to 2019, where return to play was discretionary (solely on the club doctor’s clinical judgement that the player was ready to return to competition), versus years 2021 to 2024, when the 12-day rest period was implemented. We hypothesised that the risk of further concussion and musculoskeletal injury would be notably reduced following the introduction of the mandatory rest periods (years 2021 to 2024).

## 2. Materials and Methods

AFL injury and illness data were extracted from publicly available listed injury reports on the internet covering regular season matches from seasons 2016 to 2019, where AFL clubs operated under the AFL concussion management guidelines [18], which were aligned with the then CISG consensus statement [19], and 2021 to 2024, where AFL clubs continued to adhere to the CISG-aligned graduated RTP strategy but with the additional requirement of a mandatory 12-day non-competition period before any player could return, regardless of whether symptom resolution and graduated RTP strategy criteria had been completed earlier than 12 days [14].

Ethical review and approval were waived for this study by the Health and Disability Ethics Committee NZ due to the following: (1) No direct participant or public involvement was undertaken in the collection of this data at any stage. (2) There was no recruitment or direct contact with the participants. (3) All the data was retrieved and synthesised from existing publicly available sources. (4) All the existing publicly available data did not contain personal information.

Match hours were calculated by multiplying the number of games played per season by the duration of each match. Match injury lists reported by each AFL club medical doctor at the end of matches are submitted to the AFL, with AFL media (afl.com.au) and general sports media (e.g., aflratings.com) publishing player injury data via their respective websites that are freely available to the public. The 2020 season was not included in this study due to disruption of the 2020 season from COVID-19, resulting in a shortened match season and limited matches, and also a discontinued five-day medical clearance policy. Table 1 illustrates descriptive injured players’ data collected during the time period.

### 2.1. Data Extraction Method

Source documents are web-page downloads of injury lists from common outlets including www.afl.com.au/matches/injury-list (accessed on 7 November 2025) or, where not available, www.aflratings.com (accessed on 12 November 2025) as either the raw HTML of those pages or the available PDFs of the same information. The pipeline is driven by a configurable year and round set (e.g., home-and-away rounds R1-R24 per season); for each year and round, the system resolves the appropriate source (HTML or PDF) and runs a single extraction method. We preferred direct, explicit parsing of the HTML where available in the first instance.

For confidence in data acquisition, we compared three extraction methods across all seasons on a four-round sample (rounds 1, 6, 12, and 23) of documents with manual injury counts from an audit (actual vs. extracted number of injuries per document). The methods were: (1) HTML-only parsing (analysing and converting to a structured format for analysis); (2) PDF extraction using pdfplumber (a common Python 16.108.3 library for table and text extraction from PDFs); and (3) a “progressive” method that utilises HTML when available and falls back to *Google Gemini* document intelligence to process the PDF when HTML is missing or yields very few records. Alignment was defined as the ratio of extracted injury count to manual (audit) count for each document, so that 1 indicates perfect agreement. On this verification sample, the “progressive” method substantially outperformed HTML and *pdfplumber* (mean alignment 0.98, mean absolute error 2.9 injuries per round vs. mean error 26.2 and 50.4 for HTML and *pdfplumber,* respectively).

### 2.2. Data Arrangement

Player data was deidentified and inputted into an Excel spreadsheet (Microsoft, Redmond, WA, USA) for playing season, player code, injury type/region, and round injured. Injuries listed in the reports were mapped to a five-category anatomical region schema: Head, Upper_body [shoulder/neck], Trunk_Spine [chest/abdomen], Lower_Limb [hip/legs/feet], and Other_Unknown [e.g., personal reasons, suspension]) derived from the official AFL annual injury reports.

Data cleaning involved removing repetitive injury data. For example, if an injured player was listed for the same injury across several consecutive weeks (e.g., a hamstring taking four weeks to recover), only the first report of that injury was included in the analysis. For players with repetitive *similar* injuries but no consecutive reports made, announcements separated by a minimum of four weeks were included (e.g., muscle contusion in round two, and then again in round 10).

The analysis cohort comprised players who sustained at least one injury during a given season. Players were classified by the nature of their first reported injury (Head injury-first or MSK-first), and subsequent injuries during the same season were used to determine re-injury risk. Players who did not sustain any injury during a season were not included in the risk-ratio denominators, as the analysis examines re-injury risk conditional on a first injury. Players in the 2021–2024 cohort were not excluded on the basis of RTP duration; the 12-day rule represents a minimum non-competition period, and players who returned later than 12 days remained eligible for inclusion in the subsequent-injury analysis.

### 2.3. Statistical Analysis

Risk ratios (RRs) were calculated to compare the risk of sustaining any *subsequent* injury following a first injury classified as head versus a first musculoskeletal (MSK) injury. The first injury per player was used to define exposure, with subsequent injury defined as the occurrence of at least one additional injury of any type after the first injury. Further analyses were also undertaken to measure subsequent risk for concussion, upper MSK, and lower MSK injuries.

Statistical analyses were conducted using Python 3 (Python Software Foundation, Wilmington, OR, USA) using the pandas [20], NumPy [21], and SciPy libraries [22]. For each season, a relative risk (risk ratio; RR) of sustaining a subsequent injury was calculated for concussion compared to MSK injury, with 95% confidence intervals (CI) derived utilising the log-binomial method and Fisher’s exact test for significance. For pooled multi-year comparisons, pooled fixed-effect estimates were calculated using the stratified Mantel–Haenszel approach and Cochran–Mantel–Haenszel *χ*^2^ for significance. Between-season heterogeneity was quantified using Cochran’s *Q* statistic and the *I*^2^ statistic. Data are presented as mean (95% CI), and significance was determined as *p* < 0.05.

## 3. Results

### 3.1. 2016 to 2019

During this period, there were 792 games with a total of 1584 match hours (396 h per season × four seasons). There were 3283 injuries reported with a mean incidence rate of 55.4 (95%CI 53.5 to 57.3) per 1000 match hrs. This resulted in a pooled concussion injury incidence of 6.2 [95%CI 5.5 to 6.8] and this varied from 5.1 (95%CI: 3.9 to 6.3) per 1000 match hrs (2016) to 7.6 (95%CI: 6.2 to 9.1) per 1000 match hrs (2018). Subsequent injuries of those players with a concussion resulted in a pooled injury incidence of 20.1 (95%CI: 17.1 to 23.6) per 1000 match hrs and this varied from 17.6 (95%CI: 12.0 to 24.8) per 1000 match hrs (2016) to 23.2 (95%CI: 16.9 to 31.1) per 1000 match hrs (2017).

#### 3.1.1. Risk of MSK After Head Injury

**Figure 1:**
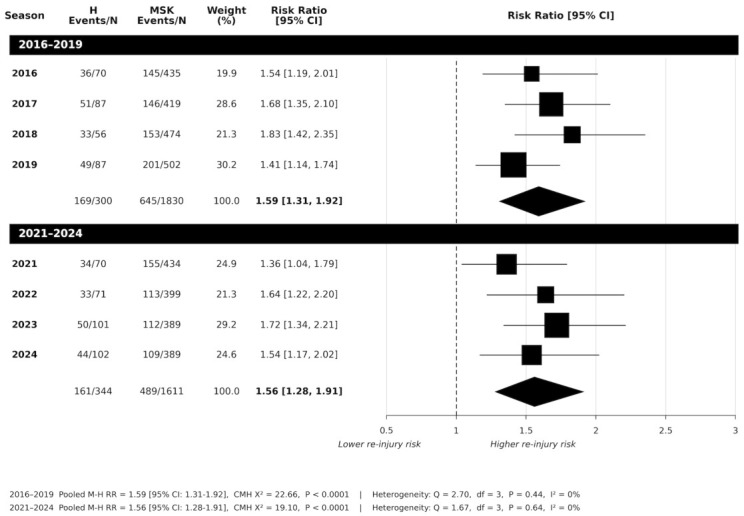
Risk of subsequent overall MSK injury: head-first injury vs. musculoskeletal-first injury across 2016 to 2019 period, and 2021 to 2024 period following introduction of the AFL 12-day stand down policy (H = head injury; MSK = musculoskeletal injury; N = total player injury).

Across individual seasons from 2016 to 2019 (see Figure 1), athletes whose first injury was classified as a head injury consistently demonstrated a significantly higher risk of sustaining a subsequent MSK injury when compared with those with an MSK-first injury (all *p* < 0.001). Pooling data across the four years (*n* = 2130) showed a statistically significant association between a first head injury and subsequent MSK injury risk, compared to MSK-first injury (pooled 2016 to 2019: RR:1.59 [95%CI:1.31 to 1.92]; *p* < 0.001). Between-season heterogeneity was not observed (*Q*_(3)_ = 2.70; *p* = 0.441; *I^2^* = 0%).

#### 3.1.2. Risk of Upper Limb Injury

**Figure 2:**
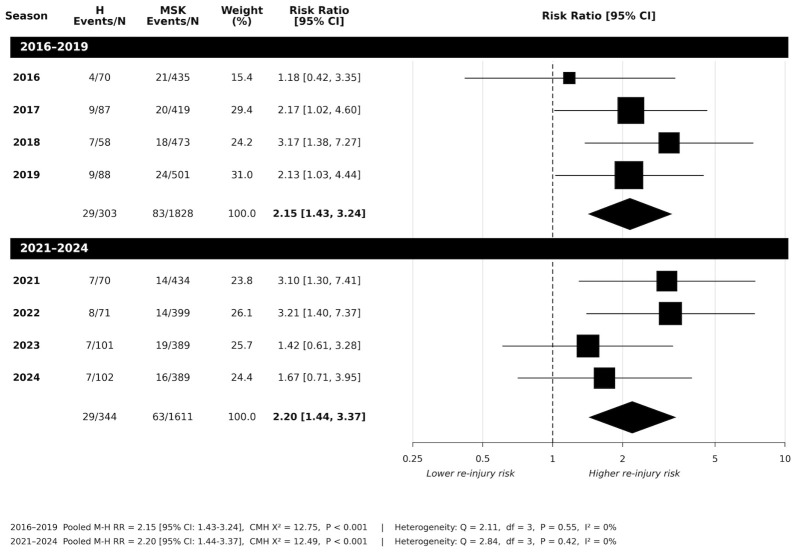
Risk of subsequent upper limb MSK injury: head-first injury vs. musculoskeletal-first injury on risk of upper body injury across 2016 to 2019 period, and 2021 to 2024 period following the introduction of the AFL 12-day stand down policy (H = head injury; MSK = musculoskeletal injury; N = total player injury).

Risk of subsequent upper limb injury is shown in Figure 2. Across seasons, there were significant increases in risk for upper limb injury for the years 2017 (*p* = 0.048) and 2018 *(p* = 0.01) but were not seen in the years 2016 (*p* = 0.765) and 2019 (*p* = 0.601). However, pooled analysis (*n* = 2131) showed an overall significant increase in risk across this period (RR:2.15 [95%CI:1.43 to 3.24]; *p* < 0.001). Between-season heterogeneity was not found (*Q*_(3)_ = 2.11; *p* = 0.550; *I*^2^ = 0%).

#### 3.1.3. Risk of Lower Limb Injury

**Figure 3:**
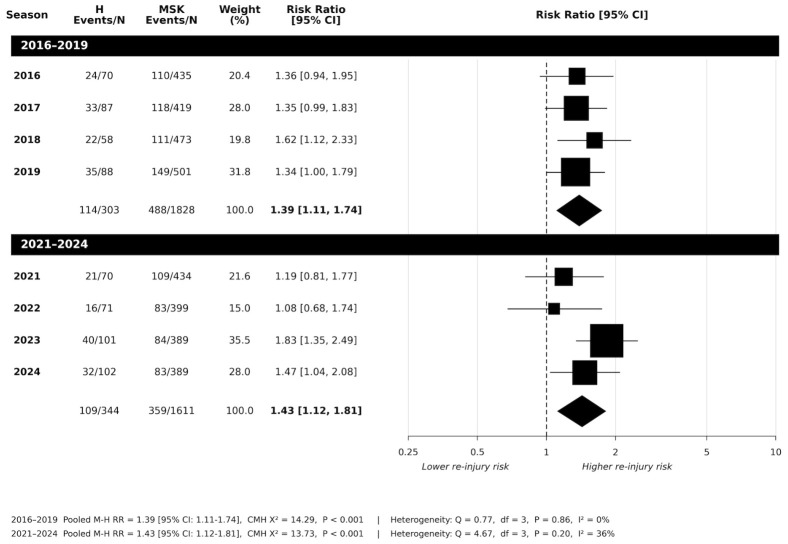
Risk of subsequent lower limb MSK injury: head-first injury vs. musculoskeletal-first injury on risk of upper body injury across 2016 to 2019 period, and 2021 to 2024 period following the introduction of the AFL 12-day stand down policy (H = head injury; MSK = musculoskeletal injury; N = total player injury).

Figure 3 shows data from the risk of lower limb injury following a head injury. Across seasons 2016–2019, only season 2018 showed statistical significance (*p* = 0.023). While seasons 2016, 2017, and 2019 showed increased risk ratios (RR:1.36, 1.35, 1.34 respectively), these were not statistically significant (*p* = 0.143, *p* = 0.073, *p* = 0.080 respectively). Pooled analysis (*n* = 1955), however, showed a significant increase in overall risk (RR:1.39 [95%CI: 1.11 to 1.74]; *p* < 0.001). No heterogeneity was found across seasons (*Q*_(3)_ = 0.770; *p* = 0.856; *I*^2^ = 0%).

#### 3.1.4. Risk of Subsequent Head Injury

**Figure 4:**
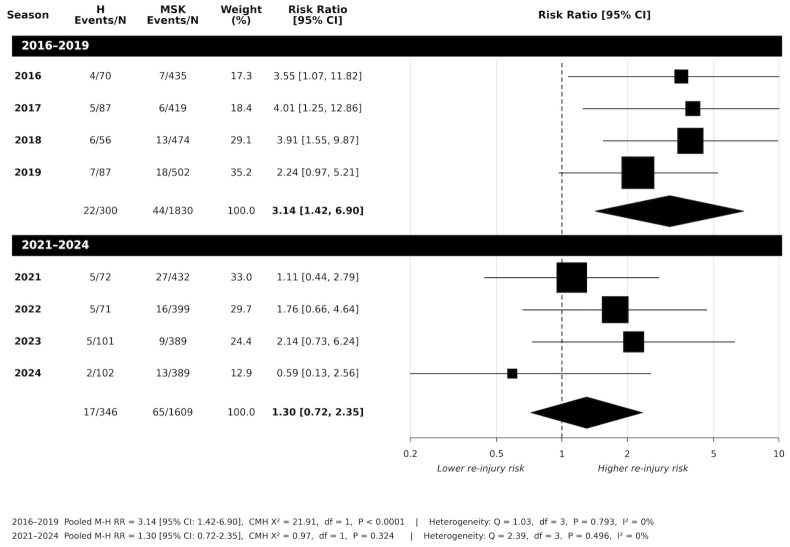
Risk of subsequent concussion injury: head-first injury vs. musculoskeletal-first injury across 2016 to 2019 period, and 2021 to 2024 period following introduction of the AFL 12-day stand down policy (H = head injury; MSK = musculoskeletal injury; N = total player injury).

Athletes at risk of subsequent head injury from seasons 2016 to 2019 are shown in Figure 4. Seasons from 2016 to 2018 showed significantly increased risk of subsequent head injury (RR range 3.55 to 4.01; *p* < 0.05). The 2019 season showed an increased risk of head injury (RR:2.24), but this was not statistically significant (*p* = 0.078). However, pooled data across 2016 to 2019 (*n* = 2130) showed a statistically significant association between a first head injury and subsequent injury risk, compared to MSK-first injury (RR:3.14 [95%CI:1.42 to 6.90]; *p* < 0.001). No evidence of between-season heterogeneity was observed (*Q*_(3)_ = 1.03; *p* = 0.793; *I*^2^ = 0%).

### 3.2. 2021 to 2024

During this period, following the introduction of the 12-day mandatory rest, there were 810 games with a total of 1620 match hours (396 h per season for seasons 2021 and 2022, and with an extra round implemented, 414 h for seasons 2023 and 2024). There were 3070 injuries reported with a mean injury rate of 51.7 (95%CI 49.8 to 53.5) per year. This resulted in a pooled concussion injury incidence of 7.2 [95%CI 6.6 to 7.9] per 1000 match hrs, and this varied from 6.5 (95%CI: 5.2 to 7.9) per 1000 match hrs (2022) to 7.6 (95%CI: 6.2 to 9.1) per 1000 match hrs (2023). Subsequent injuries of those players with a concussion resulted in a pooled injury incidence of 18.6 (95%CI: 15.8 to 21.7) per 1000 match hrs and this varied from 16.7 (95%CI: 11.6 to 23.2) per 1000 match hrs (2021) to 20.9 (95%CI: 15.5 to 27.6) per 1000 match hrs (2023).

#### 3.2.1. Risk of MSK After Head Injury

From 2021 to 2024, a head injury as the initial injury event was associated with an increased likelihood of sustaining at least one subsequent MSK injury compared with an initial MSK injury. Across individual seasons, risk ratios indicated higher subsequent injury risk following head injury (all years *p* < 0.05; see Figure 1). Pooling data across 2021 to 2024 showed a statistically significant association between a first head injury and subsequent injury risk, compared to MSK-first injury (RR:1.56 [95%CI: 1.28 to 1.91] *p* < 0.001). No between-season heterogeneity was observed (*Q*_(3)_ = 1.67 *p* = 0.640; *I*^2^ = 0%).

#### 3.2.2. Risk of Upper Limb Injury

The risk of upper limb injuries after head injury for the years 2021 to 2024 is illustrated in Figure 2, showing that seasons 2021 and 2022 showed significantly increased risk ratios in upper limb injury (RR: 3.10 and 3.21, respectively). Seasons 2023 and 2024 also showed increased risk (RR: 1.42, 1.67, respectively). However, pooled risk ratio analysis (*n* = 1955) showed an overall significant increase in risk ratios across this period (RR:2.15 [95%CI: 1.43 to 3.24]; *p* < 0.001). Between-season heterogeneity was not found (*Q*_(3)_ = 2.11; *p* = 0.550; *I*^2^ = 0%).

#### 3.2.3. Risk of Lower Limb Injury

Seasons 2021–2024 are presented in Figure 3. No significant risk in lower limb injury was identified in seasons 2021 (*p* = 0.386) and 2022 (*p* = 0.741). However, seasons 2023 and 2024 revealed significant increased risks ratios (RR:1.83 and 1.47 respectively). Pooled data (*n*= 1955) showed significantly increased risk ratios (RR:1.43 [95%CI: 1.12 to 1.81]; *p* < 0.001). While not statistically significant, between-season heterogeneity was found to be moderate (*Q*_(3)_ = 4.67; *p* = 0.198; *I*^2^ = 36%).

#### 3.2.4. Risk of Further Head Injury

Figure 4 shows the 2021 to 2024 seasons. Across all seasons, there were no significantly increased risks of subsequent head injury (all seasons *p* > 0.05). Consequently, pooled data showed no notable increased risk of subsequent head injury (RR:1.30 [95%CI: 0.72 to 2.35]; *p* = 0.324). No between-season heterogeneity was observed (*Q*_(3)_ = 2.39; *p* = 0.496; *I*^2^ = 0%).

## 4. Discussion

While extending from previous research [11], this is the first study to specifically examine the association between mandated rest periods after concussion and the risk of subsequent MSK injury in professional AFL athletes. Supporting previous systematic reviews in adult athletes across different sports [3], we found a notably increased risk of subsequent MSK injury following a concussion across both periods. Further analyses also showed that concussion increased the risk of further injury in both upper and lower limbs, which did not differ before or after the implementation of the rest period. However, while concussion risk was significantly increased in the 2016 to 2019 period, a corresponding statistically significant increase was not demonstrated in the 2021 to 2024 period.

In 2021, the AFL imposed a 12-day mandatory non-competition period for players diagnosed with a concussion to improve management and ensure full recovery of the brain after injury. This allowed us a unique opportunity to test this hypothesis and to examine whether a 12-day rest period would be associated with lower subsequent injury risk. Except for recurrent concussion risk, our hypothesis was not supported.

Overall MSK risk, which included injury to anywhere in the body, was substantially increased across both periods before and after the policy change (RR:1.56 vs. 1.59). The reported data here, while noteworthy, is lower than previously reported systematic reviews by McPherson et al. [3] and Reneker et al. [4]. This reflects the different methodological approaches to address the question across different sports cohorts, while our study investigated the risk in one sport only, utilising a homogeneous population group. However, collectively, our study supports the findings by these authors, suggesting that concussion injury increases the overall MSK risk.

Furthermore, regarding the overall MSK risk, we addressed the question of upper and lower limb injury risk following concussion. Upper limb injuries in our study highlighted an increased risk ratio (RR:2.15), reflecting previous studies investigating upper limb injury risk [5,23,24]. Moreover, we observed no change in the risk ratio (RR:2.20) after the introduction of the 12-day period. Lower limb risk ratios showed an increased risk ratio (RR:1.39), supporting similar findings from previous studies across various cohorts [24,25,26,27]. Similarly, we did not find a change in risk ratios following the introduction of the mandatory rest period.

Observed increases in MSK risk are being attributed to residual deficits in neuromuscular control and attentional deficits [28]. With direct experimental evidence lacking, the exact mechanisms remain unknown. However, evidence from neurophysiological studies suggests that impairments may stem from corticospinal impairments. For example, in one of the first studies to explore residual effects, Slobounov et al. [29] reported that movement-related cortical potentials remained impaired for up to 30 days, despite recovery of symptoms within 10 days. Similarly, recent research by Pearce et al. [9] using single-pulse transcranial magnetic stimulation (TMS) found increased cortical inhibition from motor evoked potentials lasting up to 26 days, despite symptom resolution by 12 days. Dual-task motor-cognitive deficits have also been suggested, with research showing changes in gait and speed in dual motor task performance, while in motor-cognitive performance, cognitive attention and processing speed have been shown to worsen when compared to single-task performance [28,30]. Further research is required, as a limitations of dual-task research to date may not reflect the complexities of sport skills and generate motor patterns and distribution of attention across multiple incoming stimuli during matchplay [28].

While MSK injury risk was found to be notably increased post-concussion and remained similar across both periods, this was not the case observed with concussion. Following the introduction of the policy, the pooled risk of subsequent concussion did not reach statistical significance. The wide confidence interval reflects the limited number of subsequent concussion injuries from 2021 to 2024 (*n* = 17/346), making definitive conclusions about risk compared to the 2016–2019 period difficult. Seasons 2021 to 2023 showed increases with a pooled estimate of 1.54, but with confidence intervals crossing 1.0, and season 2024 yielded a reduced estimate (RR:0.59), contributing to the overall pooled estimate of 1.30.

One of the main objectives of the 12-day non-competitive period has been to address the concerns around the management of concussion and to reduce the risk of further concussions. Previous studies have shown that the risk of concussion has been between 1.98- and 5.80-fold risk [4,31,32,33], which we found in our study (RR:3.14) from seasons 2016 to 2019, where there was no mandatory rest period. However, in the years 2021 to 2024, this association did not reach statistical significance (RR:1.30). While concussion numbers and incidence rates have not changed over both periods (2016 to 2019 and 2021 to 2024), the reduction in risk from 2021 to 2024 may be associated with the enforced rest period. As opined by McCrea et al. [17], having a period of rest (i.e., non-contact training and competition) and recovery prevents early re-exposure risk, particularly when the brain is vulnerable to the effects of repeat sub-concussive trauma in training and practices. While the elevated concussion risk observed in the 2016 to 2019 period was not replicated in the 2021 to 2024 period, the risk of neuromuscular injury remained elevated, although this distinction should be interpreted cautiously given the limited statistical power for the concussion outcome. Previous studies in concussion recovery by Pearce et al. [8] using single and paired pulse TMS have shown disparities in evoked potential changes between single-pulse neuromuscular corticospinal inhibition and paired-pulse cortical excitability. Further work to elucidate these findings is warranted.

Given that lower levels of sport now have a 21-day mandatory non-competition period, it may appear that longer rest periods for professional athletes may warrant consideration to support full recovery of the neuromuscular system [9]. Further prospective studies quantifying recurrent injury risk in lower levels of sport should be undertaken, while trials of longer rest periods in professional athlete competitions should also be considered.

### Limitations

Several limitations in this study should be taken into consideration when interpreting the results. While AFL injury data is assumed accurate, the retrospective analysis of publicly reported data carries an inherent risk of misclassification and reporting bias compared with direct access to club-level medical records. Inconsistencies were noted in concussion terminology and in the documentation of recurrent musculoskeletal injuries, where players were sometimes omitted from one weekly report and reappeared the following week. Our data are also limited to match injuries only and may differ from official AFL injury reports that incorporate both match and training exposure hours across AFL and state league levels. However, AFL clubs do not release individual-level injury data to external researchers, and the publicly reported match-week injury lists represent the only consistently available data source covering the full study period at the player level. As such, this study necessarily relies on these public sources, with the trade-off that longitudinal coverage across multiple seasons and policy eras is achieved at the cost of more granular data and direct verification of that data.

The publicly reported AFL injury data does not contain individual-level covariates such as minutes played, playing position, injury severity, prior injury history, training load, or fatigue indices. Consequently, adjustment for these variables was therefore not possible. Our analysis is positioned as a comparison of policies (pre vs. post 2020) rather than an individual-level risk model. We note, however, that homogeneity of risk estimates within each era (*I*^2^ = 0% for most primary outcomes) provides support that risk ratios were stable across seasons within each period, mitigating concerns about unmeasured between-season variation, including any small drift in mean player body mass observed across the study period. Similarly, while AFL rule changes have evolved over time, no specific rule change between the two periods has been identified that would systematically alter injury risk independently of the rest-period policy.

Per-player time from injury to RTP data were not available in the publicly reported AFL injury data for either period. While Pearce et al. [16] reported a group mean RTP of 16.2 days for 2017–2019, individual-level RTP durations could not be calculated for our cohort, and so the actual time-to-RTP between the discretionary and mandatory periods cannot be directly compared. While the CISG consensus statement has been consistent in recommending a graduated return-to-play strategy across both periods, adherence to standardised recovery protocols at the club level cannot be verified from publicly reported data.

We further acknowledge the limited statistical power for the subsequent concussion outcome, particularly in the 2021 to 2024 period (*n* = 17/346 concussion-first players). The wide confidence interval for the 2021 to 2024 pooled risk ratio is consistent with that observed in 2016 to 2019. Accordingly, the absence of a statistically significant elevation from 2021 to 2024 should be interpreted as a failure to demonstrate elevated risk, rather than as direct evidence of risk reduction.

Finally, our data only includes the AFL men’s population. The AFL Women’s competition did not start until 2017, with very short seasons. As the Women’s league has grown, rounds have increased from seven to 12. As the league matures, future studies should investigate the Women’s league, given previous research has shown sex differences with concussion and recovery between male and female athletes [34].

## 5. Conclusions

This study has reported that, prior to the implementation of the 12-day mandatory rest period, further risk of MSK and concussion injury was significantly increased. While no change in risk was found in MSK injury following a concussion across both periods (2016 to 2019 and 2021 to 2024), subsequent concussion risk was lowered during the 2021 to 2024 period compared to the elevated risk observed during the 2016 to 2019 period. While these findings are observational and do not establish causation, they raise the possibility that longer rest periods may warrant consideration in relation to all injury risk following concussion for professional athletes.

## Figures and Tables

**Table 1:** Descriptive data of year cohorts by year of collection, participant height and body mass reported by means with 95% confidence intervals (95% CI).

Year (*n*)	Player Age (Yrs) Mean (95% CI)	Player Height (cm) Mean (95%CI)	Player Body Mass (kgs) Mean (95%CI)
2016 (*n* = 543)	23.2 (22.8 to 23.5)	187.2 (186.5 to 187.9)	86.9 (86.0 to 87.7)
2017 (*n* = 541)	23.5 (23.2 to 23.8)	187.1 (186.5 to187.8)	87.1 (86.3 to 88.0)
2018 (*n* = 556)	23.4 (23.1 to 23.7)	187.2 (186.5 to 187.9)	86.8 (85.9 to 87.6)
2019 (*n* = 605)	23.6 (23.3 to 23.9)	187.2 (186.4 to187.8)	86.4 (85.6 to 87.2)
2021 (*n* = 527)	23.8 (23.4 to 24.2)	187.2 (186.5 to 187.9)	85.9 (85.1 to 85.7)
2022 (*n* = 529)	23.9 (23.5 to 24.3)	187.3 (186.6 to 188.0)	85.6 (84.8 to 86.4)
2023 (*n* = 526)	24.1 (23.7 to 24.5)	187.4 (186.7 to 188.1)	85.5 (84.7 to 86.2)
2024 (*n* = 524)	24.0 (23.6 to 24.4)	187.0 (186.3 to 187.7)	84.7 (83.9 to 85.4)

## Data Availability

Data were derived from the following resources available in the public domains afl.com.au (accessed on 7 November 2025) and aflratings.com (accessed on 12 November 2025).
